# The effect of radial shockwave on the median nerve pathway in patients with mild-to-moderate carpal tunnel syndrome: a randomized clinical trial

**DOI:** 10.1186/s13018-022-02941-9

**Published:** 2022-01-25

**Authors:** Atieh Habibzadeh, Roghayeh Mousavi-Khatir, Payam Saadat, Yahya Javadian

**Affiliations:** 1 Department of Physiotherapy, Babol University of Medical Sciences, Babol, Iran; 2 Department of Physiotherapy, Babol University of Medical Sciences, Babol, Iran; 3 Department of Neurology, Babol University of Medical Sciences, Babol, Iran

**Keywords:** Carpal tunnel syndrome, Extracorporeal shockwave therapy, Pain, Nerve conduction study

## Abstract

**Background:**

This study aimed to evaluate the short-term effect of radial shockwave on the median nerve pathway as a new model method in patients with mild-to-moderate carpal tunnel syndrome.

**Methods:**

In this randomized clinical trial, 60 patients were randomly allocated into three equal groups. The first group received 1500 shocks on the carpal tunnel, the second group received 1500 shocks on the carpal tunnel and median nerve pathways, and the third group was the control group. In all three groups, patients received conventional physiotherapy for ten sessions. In addition, patients in experimental groups received four sessions of radial shockwave. Pain and paresthesia intensity, sensory and motor distal latency were evaluated as primary outcomes. Boston carpal tunnel Questionnaire scores were evaluated as secondary outcomes. Evaluations were performed at baseline, 1 and 4 weeks after the end of the treatment.

**Results:**

Pain and paresthesia intensity and Boston questionnaire score significantly decreased in all three groups, but the greater improvement was noted in shockwave groups. Sensory and motor distal latency were only improved in shockwave groups. In terms of clinical and electrophysiological parameters, two groups of shockwaves showed similar results.

**Conclusions:**

Radial shockwave combined with conventional physiotherapy is an effective noninvasive treatment for mild-to-moderate carpal tunnel syndrome that produces greater and longer-lasting results than conventional physiotherapy alone. There were no differences observed between utilizing radial shockwave on the carpal tunnel or median nerve pathways on the palmar surface of the hand, in terms of clinical and electrophysiological measurements.

*Clinical Trial registration number* The study was registered at https://fa.irct.ir/user/trial/49490/view (20200706048028N1) in date of 08/24/2021.

**Supplementary Information:**

The online version contains supplementary material available at 10.1186/s13018-022-02941-9.

## Introduction

The compression of the median nerve as it crosses the wrist in the carpal tunnel causes median nerve mononeuropathy, also known clinically as carpal tunnel syndrome (CTS) [1]. The prevalence of CTS is approximately 8% of the world's population [2]. Symptoms of this syndrome include pain, numbness, and tingling sensation in the median nerve dermatome [3, 4]. According to the American Neuromuscular and Electrodiagnostic Medicine Association, CTS is classified into four categories: mild, moderate, severe, and very severe [5]. Noninvasive interventions such as medication, activity modification, splints, and physiotherapy are preferable to surgery, in mild-to-moderate severity of this syndrome [6].

Extracorporeal shock wave (ESW) therapy, a new therapeutic method for the treatment of mild-to-moderate CTS, has recently attracted more attention [7]. Over the last ten years, shockwave therapy has been recognized as a treatment for a variety of musculoskeletal disorders such as plantar fasciitis, lateral epicondylitis, and biceps tendinopathy [8–12]. ESWs was defined as a series of acoustic pulses with a high peak pressure, rapid pressure increase, short duration, and energy density ranging from 0.003 to 0.89 mJ/mm [13], and these were propagated in both focus and radial patterns [14]. Xie et al. reported that radial shock wave has more advantages than focused shock wave due to the large treatment area, for CTS patients [15].

Seok et al. in 2013 demonstrated that one session of shockwave therapy may be as effective as local corticosteroid injection in improving subjective symptoms in patients with mild-to-moderate CTS [16]. A systematic review study by Kim et al. in 2019 reported that shockwaves can improve subjective symptoms and electrophysiological parameters of the median nerve in patients with CTS [7]. In 2018, Atthakomol et al. demonstrated that radial shockwave can have long-term positive therapeutic effects (at least six months) in these patients [17].

In patients with mild-to-moderate CTS, there is a possibility of other accompanying pathologies, particularly distal to the site of entrapment, such as neurogenic inflammation, as well as damage to sensory and motor branches of the median nerve on the palmar surface of the hand, in addition to the nerve pathology at the carpal tunnel site [18]. Animal studies reported that low-energy shockwaves can stimulate nerve regeneration, improve nerve conduction velocity, and amplitude in rats treated with nerve autographs of the sciatic nerve [19]. Low-energy radial shockwave has also been demonstrated to reduce neuropathic pain in rats with chronic constriction injuries [20].

Previous studies have evaluated the effect of shockwave directing at the carpal tunnel site (in this study, we named it the Point method); however, there is no previous trial that has studied the effect of radial shockwave on the median nerve pathways on the palmar surface of the hand (in this study, we named it Sweep method), in patients with mild-to-moderate CTS. Therefore, this study aimed to evaluate the short-term effect of radial shockwave on the median nerve pathway as a new model method in patients with mild-to-moderate carpal tunnel syndrome.

## Methods

### Study design

**Fig. 1:**
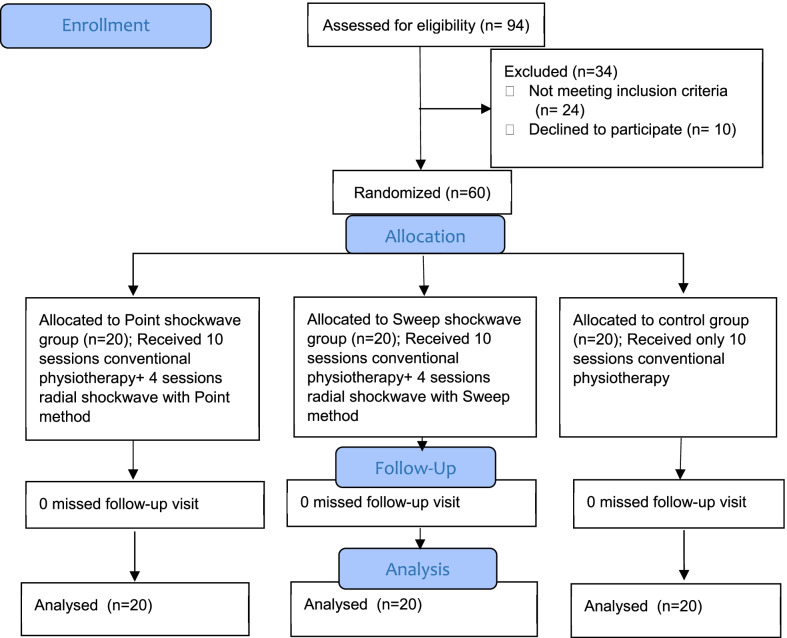
Flowchart of the study protocol

This randomized, single-blind, clinical trial was conducted in Iran at the Educational and Therapeutic Center of Ayatollah Rouhani Hospital from June 2020 to April 2021. In this study, 94 patients diagnosed with CTS were screened for eligibility; of them, 60 patients with mild-to-moderate CTS were enrolled and completed all stages of the study (Fig. 1). To determine the sample size, we use the VAS (Visual Analog Scale) [21]. The calculation of sample size was based on an alpha of 5% and a beta of 20%, with a post hoc power analysis of 0/97. Informed consent was provided by participants before enrollment. This study was approved by the Ethics Committee of Babol University of Medical Sciences with a code no: IR.MUBABOL.HRL.REC, and also registered at the Iranian Registry of Clinical Trials (IRCT) with the number 20200706048028N1.

### Randomization

These 60 enrolled patients were blocked randomized with a 1:1:1 ratio into three groups equally by an independent researcher using computer-generated randomization of study number.

### Inclusion and exclusion criteria

Patients who met the following criteria [22]: (1) aged between 20 and 60 years; (2) the presence of clinical symptoms such as pain, tingling, and numbness on the first, second, and third fingers; (3) positive Phalen’s test and Tinel’s sign; (4) pain intensity of at least 30 and maximum 60 based on visual analog scale (5) mild-to-moderate confirmation of the syndrome based on electrophysiological findings (median sensory distal latency > 3.5 ms. and median motor distal latency > 4.2 ms.). Exclusion criteria included the following [22]: (1) severe cases of the syndrome (absent of sensory and thenar motor response); (2) thenar muscle atrophy; (3) history of wrist fracture, corticosteroid injection, and carpal tunnel release surgery; (4) a systemic disease such as diabetes mellitus, rheumatoid arthritis, and severe thyroid disorders; (5) disorders that mimic CTS, such as polyneuropathy, cervical radiculopathy, and thoracic outlet syndrome; (6) cancer; (7) pregnancy; (8) history of physiotherapy treatment in the previous six months.

### Interventions

Patients were randomly divided into three groups, namely the Point shockwave group, the Sweep shockwave group, and the control group. Ten sessions of conventional physiotherapy were equally given to all three groups over 3 weeks consists of patient education, conventional transcutaneous electrical nerve stimulation (TENS) for 20 min (frequency of 100 Hz and pulse duration of 80 ms), pulse therapeutic ultrasound (US) for 5 min on the carpal tunnel area (frequency of 1 MHz and intensity of 1 W/cm^2^) [23], and patients were also instructed to wear a short cock-up splint at nights for the first two weeks of therapy and consumed one 300 mg vitamin B1 tablet per day for 4 weeks [24]. Conventional physiotherapy was done at the beginning of each session, and two groups of shockwave therapy received four sessions of radial shockwave once a week (the first, fourth, seventh, and tenth sessions), after the end of their conventional physiotherapy.

**Fig. 2:**
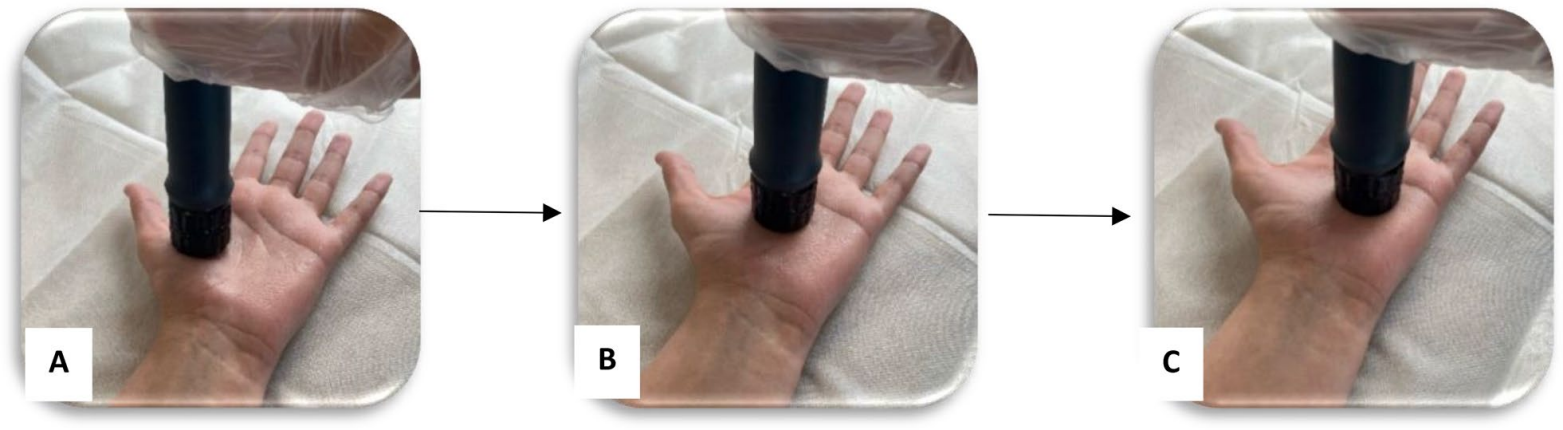
Application method of shockwave in the Sweep group; the therapy source was moved constantly back and forth, **A** between first and second metacarpals, **B** between second and third metacarpals, **C** between third and fourth metacarpals

A radial shockwave device (The Masterplus® MP100, Storz Medical, Switzerland) was used. Patients sat on a chair and bent their elbows 90 degrees and placed their forearms in a supinated position on the table. In the Point shockwave group, each patient received low-energy shockwaves with 1,500 shocks at a pressure of 1.5 bar and a rate of 6 pulses per second, perpendicular on the patient’s palm over the median nerve on the carpal tunnel after application of the coupling gel. The median nerve was localized by anatomic landmarks between the flexor carpi radialis and palmaris longus tendons, and the probe was oriented perpendicular to the carpal tunnel site during the entire procedure [22, 25]. In the Sweep shockwave group, the same parameters were used as in the Point group, but the application method was different. Thus 1000 shocks perpendicular to the patient’s palm over the median nerve on the carpal tunnel, and 500 shocks on median nerve pathways at the palmar surface of the hand were applied. The therapy source was constantly moved back and forth between the first, second, and third metacarpals (Fig. 2). Patients in the control group received only conventional physiotherapy for ten sessions.

Transient pain and redness of the skin were the most common adverse effect reported after the application of shockwave.

### Outcome measures

The investigators who assessed the clinical and electrophysiological outcomes were blinded to the group allocation, and the therapist was the independent person (assessor-blind). All evaluations were conducted before the treatment and repeated at 1 and 4 weeks after the end of the treatment by the same investigators, except electrophysiological outcomes that were evaluated before and only 1 week after the end of the treatment (the electrophysiological parameters of the median nerve were not re-evaluated 4 weeks after the treatment due to the coronavirus pandemic).

### Primary outcomes

1. The visual analog scale (VAS) was used to evaluate the intensity of pain at rest [26]. The patients were marked on a 100-mm horizontal line based on the severity of their pain intensity.
2. To evaluate the intensity of paresthesia, we also used a 100-mm horizontal line, with the words “no paresthesia” and “extremely severe paresthesia” at the opposite ends [27].
3. The median sensory and motor distal latency were evaluated according to standard protocol [28], by electromyography device (NR Sign-5000Q model made in Canada) in all patients. The surface temperature of the tested hand and wrist was maintained above 32 degrees of Celsius. Sensory distal latency (milliseconds; SDL) of the median nerve was measured by stimulating the examined wrist and recording the peak latency 14 cm away in the middle finger. Motor distal latency (milliseconds; MDL) of the median nerve was measured from the wrist to the abductor pollicis brevis muscle.

### Secondary outcomes

The valid and reliable Persian version of the Boston Carpal Tunnel Questionnaire (BQ) was used for the measurement of the symptoms severity score (SSS) and functional status score (FSS) [29, 30].

### Data analysis

Statistical analysis was performed using SPSS version 24. Demographic data was analyzed by the one-way ANOVA test for continuous data and the Chi-square test for categorical data. A one-way ANOVA was used to compare the variables of three groups followed by the Turkey HSD test. For comparing each variable before, 1 and 4 weeks after the treatment within each group, repeated measures of ANOVA was used. The significance level was set at *p* < 0.05.

## Results

Sixty patients with mild-to-moderate CTS (with a mean age of 48.98 ± 10.7 years and duration of CTS symptoms of 44.77 ± 21.5 months) completed the study in three groups. Table 1 shows the baseline demographic including age (*p* = 0.18), body mass index (*p* = 0.85), gender (*p* = 0.30), duration of symptoms (*p* = 0.83), and clinical characteristics between the three groups with no significant differences.

**Table 1:** Baseline demographic and clinical characteristics of three groups

	Point SW	Sweep SW	Control	*p* value
Age (years) (mean ± SD)	45.40 ± 11.49	50.55 ± 11.99	51 ± 7.77	0.18
Body mass index (mean ± SD)	27.66 ± 3.64	27.36 ± 3.33	28.58 ± 3.84	0.85
Gender, n (%)				
Male	2 (10%)	2 (10%)	5 (25%)	0.3
Female	18 (90%)	18 (90%)	15 (75%)	
Symptom duration, n (%)				
< 1 year	7 (35%)	8 (40%)	8 (40%)	
1–5 years	7 (35%)	9 (45%)	7 (35%)	0.83
> 5 years	6 (30%)	3 (15%)	5 (25%)	
Pain (VAS) (mean ± SD)	45.50 ± 12.34	39.50 ± 17.00	48.00 ± 10.05	0.13
Paresthesia (VAS) (mean ± SD)	52.0 ± 14.36	42.0 ± 16.73	46.00 ± 13.13	0.10
BQ (SSS) (mean±SD)	23.65 ± 5.06	21.01 ± 4.84	22.44 ± 3.96	0.23
BQ (FSS) (mean ± SD)	14.88 ± 3.84	12.40 ± 3.76	13.04 ± 2.80	0.088
SDL (mean ± SD)	4.34 ± 0.98	4.12 ± 0.97	4.40 ± 0.82	0.60
MDL (mean±SD)	4.68 ± 1.09	4.22 ± 0.97	4.50 ± 0.88	0.33

SW, shock wave; VAS, visual analog scale; BQ, Boston questionnaire; SSS, symptom severity score; FSS, functional status score; SDL, sensory distal latency; MDL, motor distal latency

Pain and paresthesia VAS and SSS significantly declined in all three groups at 1 and 4 weeks after the end of treatment, but compared to the control group, significantly greater improvement was noted in Point and Sweep groups (Table 3). In Point and Sweep groups at 4-week follow-up compared to 1 week after the end of the treatment, reduction in pain VAS (Point G: *p* = 0.163; Sweep G: *p* = 0.083), paresthesia VAS (Point G: *p* = 0.004; Sweep G: *p* = 0.163), and SSS (Point G: *p* = 0.067; Sweep G: *p* = 0.008) remained stable or the reduction process continued; however, in the control group, the above variables had significantly increased (Pain VAS: *p* = 0.002; Paresthesia VAS: *p* = 0.001; SSS: *p* = 0.021) at 4-week follow-up, compared to one week after the end of the treatment, but was still lower than baseline (*p* < 0.001) (Table 2) (Additional file 1: Figure S1–S3).

**Table 2:** Comparison of the clinical and electrophysiological parameters

	Baseline	1 week after treatment	4 weeks after treatment	*p* value	Effect size (Partial *η*^2^)		*p* value	*η*^2^
Pain (VAS) (Mean ± SD)								
Point SW	(45.50 ± 12.34)^A^	(13.00 ± 8.01)^B^	(12.00 ± 6.15)^B^	< 0.001	0.86	Group × time	0.005	0.14
Sweep SW	(39.50 ± 17.00)^A^	(13.50 ± 7.45)^B^	(12.00 ± 5.23)^B^	< 0.001	0.75			
Control	(48.00 ± 10.05)^A^	(20.50 ± 14.68)^B^	(31.00 ± 15.86)^C^	< 0.001	0.61			
*p* value	0.13	0.04	< 0.001					
Paresthesia (VAS) (Mean ± SD)								
Point SW	(52.0 ± 14.36)^A^	(18.50 ± 10.40)^B^	(14.00 ± 6.80)^C^	< 0.001	0.88	Group × time	< 0.001	0.21
Sweep SW	(42.0 ± 16.73)^A^	(13.00 ± 6.56)^B^	(12.00 ± 5.23)^B^	< 0.001	0.8			
Control	(46.0 ± 13.13)^A^	(20.50 ± 12.76)^B^	(30.00 ± 12.14)^C^	< 0.001	0.6			
*p* value	0.1	0.04	< 0.001					
BQ (SSS) (Mean ± SD)								
Point SW	(23.65 ± 5.06)^A^	(12.87 ± 2.20)^B^	(12.21 ± 1.54)^B^	< 0.001	0.88	Group × time	0.003	0.16
Sweep SW	(21.01 ± 4.84)^A^	(12.21 ± 1.87)^B^	(11.88 ± 1.65)^C^	< 0.001	0.82			
Control	(22.44 ± 3.96)^A^	(14.96 ± 3.52)^B^	(16.39 ± 3.30)^C^	< 0.001	0.66			
*p* value	0.23	0.007	< 0.001					
BQ (FSS) (Mean ± SD)								
Point SW	(14.88 ± 3.84)^A^	(9.92 ± 2.48)^B^	(9.76 ± 3.12)^B^	< 0.001	0.73	Group × time	0.006	0.14
Sweep SW	(12.40 ± 3.76)^A^	(8.88 ± 1.28)^B^	(8.48 ± 0.88)^C^	< 0.001	0.58			
Control	(13.04 ± 2.80)^A^	(10.40 ± 2.24)^B^	(10.96 ± 2.08)^C^	< 0.001	0.49			
*p* value	0.088	0.078	0.004					
SDL (ms) (Mean ± SD)								
Point SW	(4.34 ± 0.98)	(3.84 ± 0.76)	–	< 0.001	0.49	Group × time	0.002	0.19
Sweep SW	(4.12 ± 0.97)	(3.81 ± 0.94)	–	< 0.001	0.48			
Control	(4.40 ± 0.82)	(4.50 ± 0.83)	–	0.5	0.02			
*p* value	0.6	0.02	–					
MDL (ms) (Mean ± SD)								
Point SW	(4.68 ± 1.09)	(4.15 ± 1.09)	–	< 0.001	0.56	Group × time	0.002	0.19
Sweep SW	(4.22 ± 0.97)	(3.79 ± 0.87)	–	< 0.001	0.54			
Control	(4.50 ± 0.88)	(4.49 ± 0.81)	–	0.94	< 0.001			
*p* value	0.33	0.07	–					

SW, shock wave; VAS, visual analog scale; BQ, Boston questionnaire; SSS, symptom severity score; FSS, functional status score; SDL, sensory distal latency; MDL, motor distal latency

*p* value < 0.05; Letters A, B and C was used for row comparison (within groups); Similar letters indicate no significant difference

FSS significantly reduced at 1 week after the end of the treatment in all three groups with no significant difference between groups (*p* = 0.078). At 4-week follow-up, the difference was significant only between the Sweep group and control group (*p* = 0.003), so that, in the Sweep group, FSS significantly improved greater than the control group. Reduction in FSS maintained after 4-week follow-up in Point and Sweep groups, however, significantly increased in the control group compared to 1 week after the end of the treatment (*p* = 0.004) (Table 2) (Additional file 1: Figure S4).

Sensory distal latency and motor distal latency significantly improved only in Point (SDL: *p* < 0.001; MDL: *p* < 0.001) and Sweep (SDL: *p* < 0.001; MDL: *p* < 0.001) groups, and no significant reduction was noted in the control group (SDL: *p* = 0.50; MDL: *p* = 0.94) (Table 2).

In comparison, between the Point and Sweep group, no statistically significant difference was observed in terms of clinical and electrophysiological parameters (Table 3).

**Table 3:** Between groups differences at each time point

Outcome	Group	1 week after treatment		4 weeks after treatment	
		Mean diff	*p* value	Mean diff	*p* value
Pain (VAS)	Point SW vs Sweep SW	− 0.5	0.88	0.00	1.00
	Point SW vs control	− 7.5	0.04	− 19.00	< 0.001
	Sweep SW vs control	− 7.00	0.045	− 19.00	< 0.001
Paresthesia (VAS)	Point SW vs Sweep SW	5.50	0.09	2.00	0.46
	Point SW vs control	− 2.00	0.53	− 16.00	< 0.001
	Sweep SW vs control	− 7.50	0.044	− 18.00	< 0.001
BQ (SSS)	Point SW vs Sweep SW	0.59	0.73	0.34	0.89
	Point SW vs control	− 2.07	0.044	4.16	< 0.001
	Sweep SW vs control	− 2.67	0.007	4.51	< 0.001
BQ (FSS)	Point SW vs Sweep SW	1.00	0.29	1.25	0.199
	Point SW vs control	− 0.49	0.73	− 1.24	0.205
	Sweep SW vs control	− 1.5	0.06	− 2.49	0.003
SDL (ms)	Point SW vs Sweep SW	0.03	0.99	–	–
	Point SW vs control	− 0.65	0.04	–	–
	Sweep SW vs control	− 0.69	0.03	–	–
MDL (ms)	Point SW vs Sweep SW	0.35	0.23	–	–
	Point SW vs control	− 0.34	0.25	–	–
	Sweep SW vs control	− 0.69	0.02	–	–

SW, shock wave; VAS, visual analog scale; BQ, Boston questionnaire; SSS, symptom severity score; FSS, functional status score; SDL, sensory distal latency; MDL, motor distal latency

*p* value < 0.05

## Discussion

The present study revealed that both radial shockwave therapy in combination with conventional physiotherapy (TENS, US, rest splint, and vitamin B1) and conventional physiotherapy alone improved pain VAS, paresthesia VAS, SSS, and FSS in patients with mild-to-moderate carpal tunnel syndrome; however, radial shockwave could produce greater and longer-lasting results than conventional physiotherapy alone. The conventional physiotherapy modalities that reduced pain and inflammation or edema in the carpal tunnel may be responsible for the improvement in these outcomes. Some studies have found that conventional physiotherapy modalities are useful for pain alleviation and sensory complaints in patients with mild-to-moderate CTS [23, 31–34]. Previous studies have reported that splints hold the wrist in a position that minimizes pressure within the carpal tunnel, TENS activates the gate control mechanism, and ultrasound reduces inflammation of the nerve and surrounding structures, all of which result in improvements of pain VAS, paresthesia VAS, SSS, and FSS [23, 31, 33]. Since the results of this study showed that shockwave therapy combined with conventional physiotherapy improved the clinical measurements significantly more than conventional physiotherapy alone, it seems that shockwave can activate a stronger analgesic and anti-inflammation mechanism compared to conventional physiotherapy.

ESW’s clinical effect on peripheral nerves has recently gotten more attention. Several studies have attempted to use ESW as an alternative treatment for peripheral neuropathy, such as interdigital neuroma [35], stump neuroma [36], distal symmetric polyneuropathy [37], and CTS [7.10]. The exact mechanism behind the effect of ESW on peripheral neuropathy is currently unknown. However, studies have reported that ESW has an anti-inflammatory effect in musculoskeletal disorders by stimulating the production of nitric oxide [38]. Nitric oxide accumulation in the cell, which occurs when a decrease in nitric oxide is counteracted by stimulation of endothelial nitric oxide synthase in inflamed tissue, modulates NF kappa B activation, which may prevent the induction of the inflammatory process by lipopolysaccharide/interferon-gamma [39, 40]. Perineural pressure can be decreased by reducing inflammation in the carpal tunnel, and this mechanism may affect the improvement of clinical symptoms. Xu et al. compared ESW therapy and corticosteroid injection in 2019. Accordingly, results have shown that low energy radial shockwave (similar to the type and energy level used in the present study) significantly improved pain and hand function at the three-, nine-, and 12-week follow-ups in patients with mild-to-moderate CTS [25]. Raissi et al. in 2016 by utilizing radial ESW with a splint in patients with mild-to-moderate CTS demonstrated that pain intensity and BQ score significantly improved in both groups of ESW plus splint and splint alone, and no significant difference was observed between groups. According to the result of the study by Raissi, ESW with splint could not statistically reduce pain intensity and BQ scores more than splint alone. Since shockwave is dose-dependent, researchers attributed these results to the lack of appropriate dose of therapy, including the number of sessions and shocks [22]. In Raissi study, ESW with 1000 shocks was used during three sessions; however, in the present study, ESW with 1500 shocks was used during four sessions, which according to the results of the present study, improvement of pain intensity and BQ scores was significantly greater in the ESW groups than the control group.

In the control group, pain and paresthesia intensity, symptoms severity score, and functional status score significantly reduced one week after the end of treatment, but it significantly increased at four weeks after the end of the treatment; however, compared to the baseline, it showed a decrease. In the Point and Sweep ESW groups, improvement in these variables remained for 4 weeks after the treatment, which indicates a longer-term effect of ESW among mild-to-moderate CTS patients.

In term of electrophysiological parameters, Sensory and motor distal latency significantly decreased only in the Point and Sweep ESW groups, while no significant reduction was observed in the control group, which show that conventional physiotherapy modalities (Splint, TENS, and the US) fail to effectively improve the electrophysiological parameters of the median nerve. The result of the study by Xu et al. showed that low-energy radial ESW can significantly decline the median nerve sensory distal latency during three sessions in patients with mild-to-moderate CTS at 9- and 12-week follow-ups [25]. However, Wu et al. demonstrated that high-energy radial ESW (2000 shocks, a pressure of 4 bars, and a frequency of 5 kHz) failed to significantly improve sensory nerve conduction velocity [21]. Compared to the result of a study by Wu et al., our results differed because we used a low-energy and multiple-session procedure, which may have led to better results in terms of electrophysiological parameters and longer-term effects. Several studies have shown that high-energy ESW caused a significant loss of small unmyelinated nerve fibers in animals [41], whereas low-energy ESW help stimulates angiogenesis, tissue and nerve regeneration, and active Schwann cells [42, 43].

To our knowledge, the present study is the first study to investigate the effect of radial ESW on median nerve pathways on the palmar surface of the hand in patients with mild-to-moderate CTS. In this study, we utilized ESW with two different application methods, one group with 1500 shocks on the carpal tunnel, and another group with 1000 shocks on the carpal tunnel, and 500 shocks on median nerve pathways. Although there was no statistically significant difference between the two application methods of ESW in any of the variables studied, in terms of hand function, the Sweep group showed greater improvement than the Point group. It seems that utilizing ESW on the median nerve pathway in addition to the carpal tunnel may lead to better improvement in the restoration of hand function.

One of the limitations of this study is the lack of long-term follow-up and failure to re-evaluate the electrophysiological parameters of the median nerve 4 weeks after the end of the treatment due to the coronavirus pandemic. Another limitation is that both the Point and the Sweep groups received shocks to the carpal tunnel region, and only “one-third” of the shocks were given to the palmar region. It seems that the 1000 shocks at the carpal tunnel site in both the Point and the Sweep groups were sufficient to achieve positive results and the 500 shocks on the palmar surface of the hand were insufficient to achieve further improvement in the Sweep group; therefore, the future studies should use shockwave with other parameters and number of shocks on the median nerve pathways to achieve more accurate results. Since the mechanism underlying the effects of ESW on peripheral neuropathy remains unknown, it is suggested to examine and compare radial ESW with Point and Sweep methods during histological studies for future studies.

## Conclusions

According to our findings, radial shockwave combined with conventional physiotherapy is an effective noninvasive treatment for mild-to-moderate carpal tunnel syndrome that produces greater and longer-lasting results than conventional physiotherapy alone. There are no differences observed between utilizing radial shockwave at the carpal tunnel or median nerve pathways on the palmar surface of the hand, in terms of clinical and electrophysiological measurements.

## Supplementary Information

**Additional file 1: Figure S1.** The change in mean pain intensity from baseline to 1 and 4 weeks after treatment. **Figure S2.** The change in mean paresthesia intensity from baseline to 1 and 4 weeks after treatment. **Figure S3.** The change in mean BQ score (SSS) from baseline to 1 and 4 weeks after treatment. **Figure S4.** The change in mean BQ score (FSS) from baseline to 1 and 4 weeks after treatment.

## Data Availability

The datasets used and/or analyzed during the current study are available from the corresponding author on reasonable request.

## Abbreviations

CTS: Carpal tunnel syndrome
ESW: Extracorporeal shock wave
SW: Shock wave
TENS: Transcutaneous electrical nerve stimulation
US: Ultrasound
VAS: Visual analog scale
BQ: Boston questionnaire
SSS: Symptoms severity score
FSS: Functional status score
SDL: Sensory distal latency
MDL: Motor distal latency
