# Supplementary material for: The effect of radial shockwave on the median nerve pathway in patients with mild-to-moderate carpal tunnel syndrome: a randomized clinical trial
Source: J Orthop Surg Res. 2022 Jan 25;17:46. doi: 10.1186/s13018-022-02941-9 (PMC8786622; doi:10.1186/s13018-022-02941-9)
Supplement: Supplementary file 1 — Additional file 1: Figure S1. The change in mean pain intensity from baseline to 1 and 4 weeks after treatment. Figure S2. The change in mean paresthesia intensity from baseline to 1 and 4 weeks after treatment. Figure S3. The change in mean BQ score (SSS) from baseline to 1 and 4 weeks after treatment. Figure S4. The change in mean BQ score (FSS) from baseline to 1 and 4 weeks after treatment. [file 13018_2022_2941_MOESM1_ESM.docx]

**Appendix**


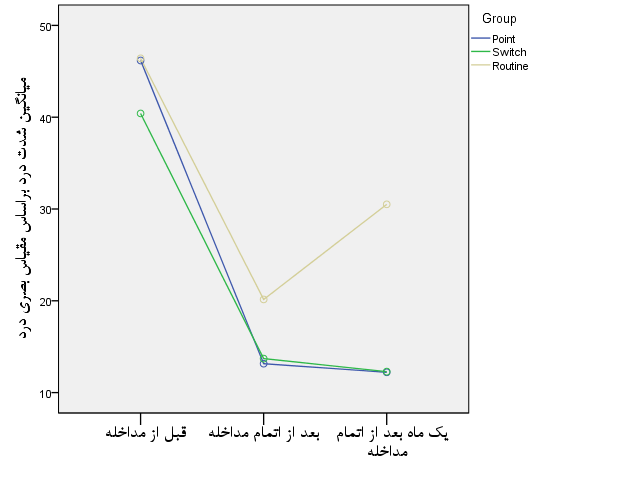


VAS (Pain)

VAS (Paresthesia)

Baseline

1week after

treatment

4weeks after

treatment

**Figure1.** The change in mean pain intensity from baseline to 1 and 4 weeks after treatment.


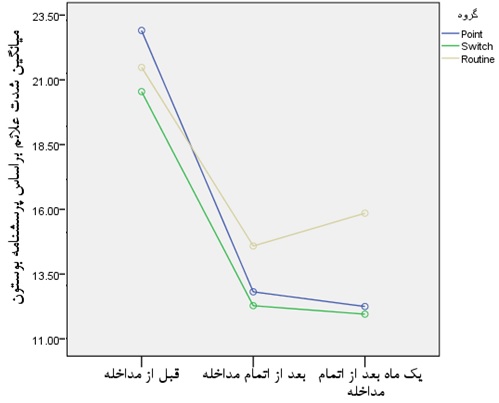


BQ (FSS)

BQ (SSS)

Baseline

1week after

treatment

4weeks after

treatment

**Figure3.** The change in mean BQ score (SSS) from baseline to 1 and 4 weeks after treatment.


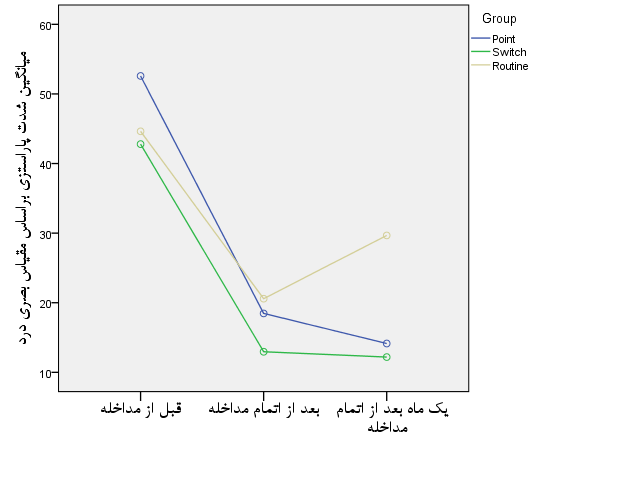


Baseline

1week after

treatment

4weeks after

treatment

**Figure2.** The change in mean paresthesia intensity from baseline to 1 and 4 weeks after treatment.


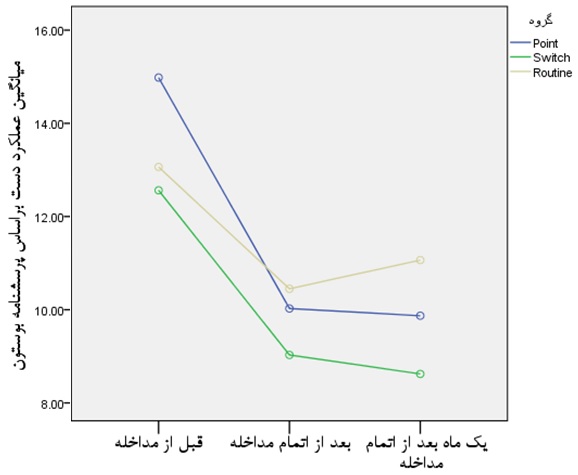


Baseline

1week after

treatment

4weeks after

treatment

**Figure4.** The change in mean BQ score (FSS) from baseline to 1 and 4 weeks after treatment.
